# Many-objective BAT algorithm

**DOI:** 10.1371/journal.pone.0234625

**Published:** 2020-06-11

**Authors:** Uzman Perwaiz, Irfan Younas, Adeem Ali Anwar

**Affiliations:** Department of Computer Science, National University of Computer and Emerging Sciences, Lahore, Pakistan; Universitat Rovira i Virgili, SPAIN

## Abstract

In many objective optimization problems (MaOPs), more than three distinct objectives are optimized. The challenging part in MaOPs is to get the Pareto approximation (PA) with high diversity and good convergence. In Literature, in order to solve the issue of diversity and convergence in MaOPs, many approaches are proposed using different multi objective evolutionary algorithms (MOEAs). Moreover, to get better results, the researchers use the sets of reference points to differentiate the solutions and to model the search process, it further evaluates and selects the non-dominating solutions by using the reference set of solutions. Furthermore, this technique is used in some of the swarm-based evolutionary algorithms. In this paper, we have used some effective adaptations of bat algorithm with the previous mentioned approach to effectively handle the many objective problems. Moreover, we have called this algorithm as many objective bat algorithm (MaOBAT). This algorithm is a biologically inspired algorithm, which uses echolocation power of micro bats. Each bat represents a complete solution, which can be evaluated based on the problem specific fitness function and then based on the dominance relationship, non-dominated solutions are selected. In proposed MaOBAT, dominance rank is used as dominance relationship (dominance rank of a solution means by how many other solutions a solution dominated). In our proposed strategy, dynamically allocated set of reference points are used, allowing the algorithm to have good convergence and high diversity pareto fronts (PF). The experimental results show that the proposed algorithm has significant advantages over several state-of-the-art algorithms in terms of the quality of the solution.

## Introduction

For multi-objective optimization [1] decision-making is based on the multiple criteria. To solve the multi-objective problems (MOPs) [2], there is a well-known family of meta-heuristic based algorithms like MOEAs [1], Multi-objective Particle Swarm Optimization (MOPSOs) [1,3] and multi-objective bat algorithms (MOBATs) [4]. MOEAs achieve the pareto-front (PF) approximation in a single run with the use of their own population-based property [2]. The qualities of the solutions are discriminated based on pareto dominance relation [1–2,5]. Consequently, MOEAs, MOPSOs and multi-objective bat algorithms can be characterized accordingly. Non-dominated sort genetic algorithm II (NSGA-II) [6], Strength pareto evolutionary algorithm 2 (SPEA2) [7], Speed-constrained multi-objective particle swarm optimization (SMPSO) [8] and multi-objective bat algorithm [4] use different variant of the pareto dominance relationship [5,9–10]. Moreover, these algorithms are known as Pareto dominance-based algorithms. MOPSO, MOEA and MOBAT [3] have obtained the preferable results using dominance relationship for MOPs up to three objectives [5,11] but these algorithms work inefficiently when more than three objectives are optimized simultaneously [5,10,12–13]. In MaOPs, more than three distinct objectives are optimized. [5,12,14].

Because of the higher number of objectives, the likelihood of the solution being dominated by the other solutions in the population drop sharply in MaOP [14]. So, the dominance criterion relation does not work to calculate the preference between the solutions. Hence, the guidance of the non-dominated solutions in the search space degrades to critical level. The selection pressure to the PF is badly affected by this attenuation in the dominance discrepancy. So, the convergence is sharply reduced [12,15]. In some case it performs as worse as the random search algorithm [12,15–17].

The downside of the MaOPs over the MOPs is because of the increased number of objective functions. Moreover, the non-dominated solutions increase exponentially [18] and this led to large population to the external archive. Consequently, evolutionary multi-objective optimization (EMO) researchers are working to find out the answers to these problems.

The several approaches are presented to get good convergence and diversity in Pareto-dominance based algorithms for MaOPs [5,19]. The several approaches include: relaxed dominance-based approach (adjusted Pareto-dominance relation), Adjusted of the density estimation (diversity-based approach), The reduction-based approach, Aggregation function, Using reference information and an approach in which solutions are guided based on of the reference set in the search space. In the above-mentioned approaches, the first two approaches can guide the population to region close to PF, but the adverse effect of this approach is that the diversity is not maintained. Although, the final output solutions are very close to PF, but they cover only small portion of the PF [5,20]. The third approach is a substitute to the Pareto dominance relationship, as the superiority of the approximation set is calculated by using an indicator function [11]. Moreover, for pareto dominance, monotonic operator is used, and quality indicator is hypervolume [14]. However, the issue is that the computational cost to compute the hypervolume is very high, that confines its application in MaOPs [5,21]. In the approach of dimensionality reduction, the first part is to remove the redundant and correlated objective from MaOPs [14]. Moreover, this approach transforms the large number of objectives into small number of objective while maintaining the same PF. Furthermore, in the next phase the selected algorithm (compact version of the original problem) is optimized. However, the issue arises when dealing with the MaOPs with no redundant objectives or after removing the redundant objectives problem, it remains a MaOPs. These cases still not yield the promising results to get a good approximation of the PF. In the aggregation function we must select a proper aggregation function, which is suitable to our problem [21,22]. However, selecting this function is a drawback in this approach. As, this approach converge the solutions to a small portion of the PF [14]. As this approach converges to a portion of PF, it is totally inefficient and useless. Moreover, the use of preference information approach could be used to give us the approximation of the PF, So the issue of exponential growth in the amount of non-dominated solutions can be addressed [5,14]. As this approach, does not focuses to approximate the whole PF but only the portion of the PF, so this approach focuses on the region of the PF, which is interested to the decision-maker. However, the disadvantage with the approach is the pressure of making good decisions, as the whole process depends on the decision-making [21]. Additionally, most of the time it is convenient to get the full approximation in the PF. In conclusion, each one has a drawback in the terms of having gotten better diversity or convergence, or they are computationally expensive, or they are not consistently working on practical problems in the MaOPs. By understanding this, one way to solve MaOPs is with the use of reference set based methodology. Algorithms based on the reference sets have more success to get the good approximation of PF and remain a research challenge [9]. The evaluation of solutions uses the reference set and then select the non-dominating solutions to get the good convergence and high diversity. This approach is somewhat based on the preference-based approach, but we have to set preferences to get the full approximation of the PF.

This paper presents the MaOBAT algorithm which uses reference point approach to solve the MaOPs. The main reason of using BAT based technique is that it converges rapidly to PF and gives good approximation of PF in MOPs as Bat algorithms are based on swarm intelligence and inspired from the echolocation behaviors of bat. Echolocation works as type of sonar; a loud sound is emitted, and echo returns after that sound hit the object. It is an important factor that bats use to identify distances, obstacles and the difference between food and prey [23]. Moreover, it also allows them to hunt in complete darkness [23]. The combination of echolocation with swarm intelligence enhances the properties of swarm-based algorithms so this thing makes BAT algorithm little more effective than the swarm-based algorithms in some of the scenarios. The fundamentals of the MOEAs and MOBATs are different from each other, so we cannot use the result and conclusion of MOEAs cannot implies anything for MOBATs. In fact, the MOEAs converge slowly to PF but BAT based algorithms converge fast in MaOPs as they converge fast in single objective optimization. Bat-based algorithms perform better than PSO-based algorithms in some problem specific scenarios [24, 25] as different studies suggest. For instance, a comparative study between BAT algorithm and PSO algorithm is performed and radial basis function network (RBF) is trained in order to classify types of benchmarked data. It is seen that BAT algorithm performed better than PSO algorithm in terms of improving the weights of RBF network and to accelerate the training time and to get good convergence of optimal solutions, which led to increase in the network efficiency and reduced falling mistakes and nonoccurrence [24]. Moreover, in another study, a comparison of algorithms for training feed forward neural networks is done. Two gradient descent algorithms (Backpropagation and Levenberg-Marquardt), and three population-based heuristic: Bat algorithm, Genetic algorithm, and Particle Swarm optimization algorithm are used for testing. Bat algorithm outperforms all other algorithms in training feed forward neural networks [25]. These studies encourage to use BA in further experiments and in further real-world applications. The benefit of the using bat algorithm is to obtain solutions based on population and local search-based algorithms. This combination gives us global diversity as well as local rigorous exploitation, which is important for metaheuristic algorithms. So, Bat algorithm is the combination of PSO and local search, which further uses pulse rate control and loudness [26]. By adapting the approach of reference sets, the MOBATs are used for the MaOPs and provide a good balance in diversity and convergence which is the main issue in the MaOPs. The main purpose of the paper is to improve the many-objective algorithm result, by implementing a new bat-inspired algorithm for many-objective optimization problems by using reference set approach to get good convergence and diversity.

The organization of the paper is as follows. Section 2 describes the Literature Review. Section 3 presents the proposed strategy for many-objective BAT-algorithm. Section 4 gives the brief explanation of state-of-the-art algorithms, which are used for the comparison. Section 5 gives introduction to test problems, parameter settings and the experiment results. conclusion and future work are presented in Section 6.

## Literature review

In literature, to improve the convergence of the MOEAs, MOPSOs and MOBAT, the relaxed form of the dominance relationships is proposed by some authors, which increase the selection pressure towards the PF. In the following section, the representative work is reviewed and then the inspiration for our work is elaborated.

Sato et al. [5] floated the idea of modified dominance relationship and it enhanced the performance of the NSGA-II. Moreover, the relationship is known as Control of Dominance Area of Solutions (CDAS). Furthermore, De Carvalho and Pozo applied CDAS on two different MOPSOs, namely sigma MOPSO and SMPSO. However, because of this updated relationship the divergence is affected badly as the selective pressure towards the PF is increased.

Mostaghim et al. [16] in 2008 proposed two aggregation methods, the first is weighted sum approach and the second one is distance-based ranking. Moreover, it is shown that for some problem the distance-based approach with MOPSO outperformed NSGA-II and random search up to 20 objectives. However, the analysis is done for the convergence, but the diversity issue is still remaining.

In order to manage both convergence and diversity simultaneously, Garza-Fabre et al. [15] provided two aggregation-based MOEAs focusing on the use of fitness assignments to emphasize convergence called the clustering elitist genetic algorithm (CEGA) and to provide an explicit system for promoting diversity called the multi-directional fitness assignment (MDFA), but it still has issues as it converges toward the small part of the PF.

Deb and Jain in 2013 [27], proposed the reformation to NSGA-II based on the reference set approach to solve the MaOPs and its named as (Non-dominated Sorting genetic Algorithm-III) NSGA-III [27]. Moreover, in NSGA-III [27], the reference set approach is used, and it showed good convergence and diversity simultaneously. In addition, the NSGA-II crowding distance is substituted by clustering operator, in which each member of the population is connected with one of the well-distributed reference points in order to achieve excellent diversity.

Figueiredo et al. [28], in 2016, used the same reference set based approach in MOPSO to solve the MaOPs with good convergence and diversity. Moreover, they proposed some more important difference in accordance to the PSO algorithms. The Many Objective Particle Swarm Optimization (MaOPSO) [28] uses the external archive to save best non-dominant solutions which can be used in the later iterations for the selection of the leaders as needed in the PSO algorithms. Another significant distinction is that MaOPSO utilizes density and pareto dominance data to move the particles towards the PF. However, no unique reproduction operator is used by NSGA-III [27].

Zhihua et al. [29], proposed an improved version of NSGA-III, in which a new selection and elimination operator is used. At first, a selection operator is used to locate the reference point with the minimum niches count, and then one individual is selected with the shortest penalty-based intersection distance. Secondly, a reference point with the maximum niche count is defined, and the elimination operator removes one individual with the longest penalty-based boundary intersection distance.

Multi-objective and multidisciplinary in nature demand coherent optimization algorithms, especially for the engineering optimization problems as they are considered as complex constrained problems. To fix this, Xin-She Yang introduced an invariant of bat algorithm known as MOBAT [3]. Yang enhanced the bat algorithm for solving global and nonlinear optimization problems.

Moreover, multi-objective bat algorithm has been used to solve many real-life applications. Arash et al. [30] proposed a multi-objective Bat algorithm for optimizing the cost for allocating human resources to an emergency hospital. Anindita et al. [31] proposed a bio-inspired algorithm for solving different aspects of wireless sensor network (WSN) like optimal routing, clustering, dynamic allocation of motes, lifetime optimization and the energy problem.

Learning to rank is an important task in Information Retrieval (IR) and multi-objective optimization algorithms have shown success in solving these kinds of problems. Tie-Yan Liu [32] provided a comprehensive overview of learning to rank for IR and categorized the existing learning-to-rank algorithms into three approaches. A detailed review of these approaches, their relationship with loss functions and IR evaluation measures are presented. A ranking model is constructed using data, this task to construct model is known as Learning to rank. Li et al. [33] proposed a multi-objective optimization model of the robust learning to rank (LTR) and this model is helpful in sorting objects according to the importance, choice and relevance. [32, 33]. The algorithm proposed in our paper can be used to solve the problem of learning to rank.

Recently, Li et al. [34] proposed a dandelion algorithm (DA), which shows excellent results in solving optimization problems, however, it converges slowly and can easily get trapped in local optima. In order to overcome these problems, Zhu et al. [35] proposed a dandelion algorithm with probability-based mutation. Levy and Gaussian mutations are used interchangeably, and it showed better results than standard DA.

Moreover, with promising results of the reference-based approach with MOEAs for handling the MaOPs, the adaption made in MOBAT using reference set approach to solve the MaOPs to achieve good convergence and diversity in more efficient manners. Additionally, this approach uses the archive to maintain best Bats as to select the leaders.

## Multi and many objective Bat algorithms

In this Section, multi and many objective Bat algorithms are discussed. Multi-Objective optimization problems are those optimization problems in which there are two or three objectives, which need to be optimized and those problems having more than three objectives are categorized as Many-objective optimization problems.

The multi-objective bat algorithm and the proposed many-objective bat algorithm are described in this following chapter.

### Multi-objective Bat algorithm (MOBAT)

Multi-objective and multidisciplinary problems in nature demand coherent optimization algorithms, especially for the engineering optimization problems as they are considered as complex constraint problems. To fix this, Xin-She Yang introduced an invariant of bat algorithm known as the MOBAT [3]. Yang enhanced the bat algorithm for solving global and nonlinear optimization problem. MOBAT is first tested on various subsets of trial functions and then it is used to give solution of multi-objective problems involving welded beam design problem. MOBAT shows promising results as compared to existing multi-objectives algorithms [2]. For MOBAT, there are number of challenges needed to be dealt with. Firstly, the multi-objective problems are quite complex and arduous to solve. As, there are no unique best solutions for a multi-objective problem, so algorithm must find a non-dominated approximated solution for the true PF. Secondly, it must guarantee that numerous PF points are distributed evenly on the front and that the algorithm works for multi-objective design issues without extra circumstances such as weighted sum methods to combine various objectives into single objectives. For the real-world optimization design problems, like engineering problems for which Yang designed MOBAT, there is mostly uncertainty and noise in the working solution.

The primary problem in multi-objective bat is to approximate or approach the optimal Pareto fronts and objectives. The bat inspired algorithm must be modified enough to cater the multi-objectives of the design problems in proper manner. In this algorithm as per Pareto optimality rule a vector v = (v1,…, vn)T is dominated by the solution space vector u = (u1,.., un)T ∈ F if and only if ui ≤ vi for ∀i ∈ {1,…,n} and ∃i ∈ {1,…,n}: ui < vi. According to this equation, it can be said that all corresponding components of (u) are equal or smaller than all components of (v) with an applied condition that at least one component of (u) should necessarily be smaller. From this we can drive dominance relationship equation ≼ i.e. u ≼ v ⇐⇒ u ≺ v ∨ u = v. From this it can be said that for finding the solutions of maximization problems, the dominance relationship sign ≺ can be inverted with ≻. A non-dominated solution point x* ∈ F can be approximated if no solution can be seen in the solution space that dominates it. The Pareto front (PF) can be represented with this equation PF = {s∈S ∃/s ∈S: s ≺s} and PF can be written in the of Pareto optimality set in search domain can be written as:

$${PF}^{*}=\{x\in F|∄x^{\prime }\in F:f(x^{\prime })≺f(x)\}$$ (1)

In the MOBAT algorithm the signal objective (f) can be found from the multi-objectives (f_k_) as the weighted sum of all multi-objectives, where the summation of all weights is equal to 1. The weighted are approximated randomly from a uniform distribution so that weights can have the required diversity to approximate the PF. If there are multiple objective functions from 1 to k, where k is the quantity of objectives of the problem, then f_1_(x),…, f_k_(x) are the objective functions given the initial feature set x = (x_1_,…, x_d_) T.

**Algorithm 1:** Framework of the Multi-objective BAT

1: Initialize the Bat algorithm population with its parameters

2: Weight vectors for all objective functions are generated, where the sum of all weights is equal to 1.

3: Execute the algorithm for maximum iterations, which is the hyper-parameter of MOBAT.

4: In every iteration the new solution for each objection function is generated by frequency and position updating equations.

5: A single objective is estimated by weighted sum of the multiple objective functions.

6: New solutions are also estimated by random walk and flying randomly, and best ones are accepted out of them.

7: After each iteration, the ratio of pulse emission and average loudness of bat algorithm is increased.

8: After each iteration, the bats are ranked as per weighted single objective function (*f*) and current best solution is found.

9: At the end best non-dominated solution is estimated and it is post processed, if required.

### Many-objective Bat algorithm (MaOBAT)

The proposed algorithm is named as Many-Objective BAT Algorithm and that is summarized in Algorithm 2. In this algorithm, the uniformly distributed random bats are generated as the initial population. After this, the algorithm evaluate bat using problem specific evaluation function. To save non-dominated solutions, MaOBAT has an external archive (A_t_). Initially Archive (A_0_) is empty. After that, reference points are generated and then distributed uniformly, which are used to compute the social leaders based on discrimination of solutions in the external archive. In each iteration algorithm chooses the cognitive and social leaders from the external archive and update particle position and velocity to move in the decision space. In the next step of the iteration, the polynomial mutation is applied to 15% of the particles. And in the final step of the iteration, pruning of the external archive is done so that the size of the external archive doesn’t overflow than the maximum size (N). The iterations are repeated till the maximum number of iterations (t_max_).

A fitness function is defined by the algorithm, based on that function the discrimination between particles is done. Based on the fitness values, the choice of the social leaders and the pruning (Selection of the best non-dominated particles in the archive) of the external archive is done. The explanation of different components of the Algorithm 2 is discussed in following sections.

#### Fitness method

It mainly focuses on two things, diversity measure to get good diversity and convergence measure to get the high convergence. To calculate these two measures, a method based on the reference points is presented. The density of the particle is then calculated by making the cluster of these particles against reference points.

#### Computation of the reference points and hyperplane

To emphasize on the convergence and well distribution of the particles to the PF, the algorithm uses the set of the reference points. Moreover, the method to get the reference point is recommended by Das and Dennis [36]. Hyperplane has well-distributed set of points in the objective space and it makes equal angle with each axis and lies in the first quadrant. A factor (p) is used here which decide in how many distributions each axis is going be divided. The entire number of reference points is formed using the given formula. $H=c_{p}^{m+p-1}$, where m is the number of objectives. For instance, with three objectives (m = 3) and p = 4, the number of reference points are 15. Moreover, the reference points on the apex of the triangle are (1,0,0), (0,1,0), (0,0,1) and these reference points are unitary reference points.

**Algorithm 2:** Framework of the MaOBAT

1: Create random initial population P0 of bats of size N

2: Take the empty initial archive A0 = ∅

3: Take the initial set of extreme point *Z*0 = ∅

4: Compute the set of reference point (∧)

5: for itr = 1 to itr_max do

6: Evaluate the of bats in P*itr* using MaOP evaluation function

7: Update the A*itr* with Aitr-1 ∪Pitr-1

8: Select the non-dominated solution from the Archive

9: Compute the ideal *Z*min from A*itr*

10: Calculate the extreme point *Zitr* from *Zitr*-1 ∪ *Aitr*

11: Using the extreme point *Zitr* to construct the Hyperplane

12: Using the Density operator calculate the density measure μ*s* of each solution *s* ∈ *Aitr* for the reference set ∧

13: Using the Convergence operator calculate the convergence measure ρ*s* of each solution *s* ∈ *Aitr* for the reference set (∧)

14: For each bat *i*∈*P*_*itr*_ calculate the social leader *l*_*i*_∈*A*_*itr*_ using density and convergence measure

15: Prune the external archive if necessary

16: Update the Velocities of bats in P*itr*

17: Update the Positions of bats in P*itr*

18: Apply the random walk on each bat.

19: Update the pulse emission rate (*α*_i_) and loudness reduction (*γ*_i_) for each bat *i* ∈ P*itr* in the population if the bat become better than the current bat after random walk.

20: end for

21: return A*itr_max*

#### Translation of the solutions

The ideal point (fi*) for each objective is the minimum value point. If the ideal is given than we use it as it is, otherwise, it calculates by taking the minimum value for each objective in the external archive. After selecting the ideal point, subtract it from each member of the external archive *fi*′(**x**) = *fi*(**x**) − fi*

Every objective has an ideal point (fi*) which is the minimum value of that objective. The objective is denoted by (i) and f* represents idea point.

#### Density operator

To get the density operator, all the bats from the (*A*_*t*_*)* are projected on the Hyperplane and each bat assigned to a reference point on the minimum perpendicular distance basis. For every bat, a cluster of bats are associated with each reference point. After computing the clusters, the density operator of each bat i.e. the number of bats attached with the reference point to which that bat attached is computed. For example, if the bat attached to the reference point j are φ*j* = {*a*,*b*,*c*}, the density measure of all the bats (*a*,*b*,*c*) is 3.

#### Convergence operator

To get good convergence, convergence operator is used. The ASF of the bat (i) of external archive with respect to that reference point to which it is associated is calculated. The convergence measure of the bat (i) is the ASF value of that bat. Mathematically it is as follows: ρ*i* = *ASF* (**x***i*, λ*j*), where *i* belongs to φ*j*.

#### Pruning the external archive

The bats in the external archive plays a vital role in the process of updating pulse emission rate and loudness. For each bat to maintain the external archive properly is quite essential in the algorithm. Updating external archive at each step is explained as follows. Firstly, non-dominated solution are added to the external archives and if the added bat is dominated by any of the bat in the external archive then remove this bat from external archive, otherwise add this bat in external archive and remove the bat of the external archive which are dominated by this bat. Moreover, if the size of the external archive increases from the maximum size, then remove the bats based on the density measure but if the two bats have the same density measure, then the convergence measure is used to differentiate between the particles. After removing the bat, the density measure for bats, which are associated with that reference point, to which removing particle is attached are decremented by one. This elimination step is being done until the external archive reach to the maximum archive size.

#### Update the loudness and rate of pulse emission

Loudness and rate of pulse emission of the bat is updated using the random walk on each bat around its leader. The equation is:

$$x_{new}=x_{old}+\epsilon \;A^{t}$$ (2)

where *ϵ* is a random value from [–1,1] for each bat and *A*^*t*^ is the average loudness of all bats for specific timestamp, if the random value from [0,1] is less than the rate of pulse emission.

In this process the leader of the bat is taken and if the rate of emission is less than the random values, the leader is updated using the given equation:

bat[i]=leader[i]+random(0,numberofdecisionvariable)*0.001 (3)

Using random flying the new bat are created. Moreover, if the fitness of the newly created bat is better than that leader and random value (which is used above for the comparison with the rate of pulse emission) is less than the loudness of that bat, then previous bat is replace by new bat solution. For the fitness comparison, if the new bat dominates then we simply replace it with the old bat but if they are incomparable than we see which bat from these two bats is near to ideal solution. The solution with the least distance from ideal point is selected. This method encourages convergence as we seek to reduce the distance from the ideal point. In the last step, if the new solution replaces the old solution that we update the pulse emission rate (*α*) and loudness reduction (*γ*) with the following equation:

$$A_{i}^{t+1}=\alpha {A_{i}^{t}}^{}$$ (4)

$$r_{i}^{t}=r_{i}^{0}[1-\exp\left(-\gamma t\right)]$$ (5)

Alpha and gamma are constants which represents pulse emission rate (*α*) and loudness reduction (*γ*) and respectively. In fact, alpha is similar to the cooling schedule in simulated annealing [37].

#### Computational complexity of one generation of MaOBAT

Initially there are some initializations (archive, population, extreme and reference points) so the computational cost for them are O (n) (line 1 to 5 in Algorithm 2). Major computations are done in each iteration of the Algorithm 2 so for that we do line by line analysis to have to proper computational analysis. Selection of non-dominant solutions needs O (N log ^M −2^ N) computations (line 8 in Algorithm 2) [38]. Selection of the ideal point (line 9 in Algorithm 2) requires a total of O (MN) computations and the identification of extreme point (line 10 in Algorithm 2) needs O (M^2^N) computations. One matrix inversion of size M×M, requires O (M^3^) operations for the determination of intercepts to construct Hyperplane (line 11 in Algorithm 2) [27]. For calculating the density measure (line 12 in Algorithm 2) and convergence measure (line 13 in Algorithm 2) of maximum 2N population members to H reference points requires O(MNH) computations. Prune external archive (line 14 in Algorithm 2) takes O(NH) computations. Properties like loudness, rate of pulse emission, velocity and position are updated in computation bounds of O(NM). In all our calculations, we have considered H almost equal to N and N greater than M. Considering all of the above computations, the Computational Complexity of One Generation of MaOBAT is O(N^2^M).

Moreover, the time complexity of MaOBAT is asymptotically equivalent to time complexity of NSGA-III.

#### Comparison of multi and many-objective bat algorithm

In multi-objective Bat algorithm problems arises when the objectives increase, as the convergence and diversity is compromised. Moreover, the proportion of non-dominated solutions in a randomly chosen set of objective vectors becomes exponentially large with the increase in number of objectives, so there is not much room left for creating new solutions and it slows down the search process. Through many-objective Bat algorithm, all the above problems are tackled as it uses reference point approach to solve the MaOPs by converging rapidly to PF and by giving good approximation of PF. Points corresponding to each reference point can be emphasized to find a set of widely distributed sets of Pareto-optimal points [27]. As the reference points are widely distributed throughout the normalized hyperplane, the solutions obtained are also likely to be widely distributed on or near the Pareto-optimal front. The goal in such a many-objective optimization is to find Pareto optimal points, which are in some way closest to the specified reference points [27].

#### Analysis of bat algorithm

Exploration (diversification) and exploitation (intensification) are two important components of a metaheuristic and there is a need to maintain an appropriate balance between them to find a near global optimum [39, 40]. Exploration property of an algorithm helps to explore unknown and new regions of the search space by generating diverse set of solutions focusing on the search at global level [41, 42]. On the other hand, exploitation capability of an algorithm exploits the information from the solution at hand and aims to improve it by searching in the local region of the search space [41, 42]. The bat algorithm exhibits the properties of both population-based methods and local search [43]. It is based on PSO [44], and uses two components, which are simulated annealing and random walk direct exploitation heuristics (RWDE) [45]. Simulated annealing heuristic [37] introduces diversity in the population and enhances the explorative capability of the bat search. RWDE [46] is connected with the exploitative capability of a bat by exploring the local region of the search space to improve the solution at hand. Exploration and exploitation in a bat algorithm are controlled by two parameters: the loudness and the pulse rate, respectively [46]. The reason of bat algorithms generating good results is due to the combination of echolocation with swarm intelligence as it enhances the properties of swarm-based algorithms in some of the scenarios. During search, the loudness and the rate of pulse emission of bats have to be updated accordingly as the iterations proceed as these characteristics help to find the optimized solutions in the better way. In early iterations, the loudness is high, which helps in exploring different regions of the search space. On the other hand, in later iterations of the search, the rate of pulse emission is increased in order to exploit the promising region of the search space.

Typically, the loudness decreases, and the rate of pulse emission increases as the bats approach towards the near-optimal solution, and this mimics the behavior of a bat when it detects and localizes a prey [45]. To control the dynamics of a swarm of bats, these parameters are tuned to maintain an appropriate balance between exploration and exploitation components of the algorithm to find the near-optimal solution.

## Many-objective evolutionary algorithms for testing MaOBAT

In this section the NSGA-III [27] and MaOPSO are explained. The performance of the MaOBAT is compared with two algorithms MaOPSO [28] and NSGA-III [27]. The brief summary of these algorithms is given below:

### NSGA-III

In NSGA-III, the initial population is created using randomly generated solutions which are also uniformly distributed. After this, the algorithm evaluates solutions using problem specific Evaluation function. Then, the offspring are generated with mutation and recombination. Then, combine the population and offspring to make the fronts based on pareto dominance relationship as done in the NSGA II [6]. Then, considering the population size, add these fronts one by one to next generation. After adding each front check whether the size of next generation is more or equal to population size. If it is equal than move to next iteration and if the algorithm hasn’t reached to the maximum specified iteration then calculate how many solutions are required to be added from the last added front and then uniformly distribute reference points are generated, which are used to normalize all the solutions. The solutions are connected with each reference point on the basis of a minimum perpendicular distance from each reference point. The next step is calculating niche count. On the basis of the niche count, without the final selected front, add solutions from the final front into the next iteration one by one until it reaches to population size.

### MaOPSO

In this algorithm [28], the uniformly distributed randomly generated particles are generated in the initial swarm S_o._ After this the algorithm evaluate particles using problem specific evaluation function. To save the non-dominated solutions MaOPSO has external archive (A_t_), initially the Archive A_0_ is empty. Then, uniformly distributed reference points are created, in order to compute the social leaders based on the discrimination of solutions in the external archive. After this, it works iteratively, in each iteration the cognitive and social leaders are updated from external archive and then these leaders are used to update position and velocity of particle to move in the decision space. In the next step of the iteration, to the 15% of the particles, polynomial mutation is applied. In the final step of the iteration, pruning of the external archive is done so that the external archives size does not overflow than the maximum size N. These iterations are repetitive until the maximum iterations (t_max_). The algorithm defines a function for fitness and on the basis of the that function, the discrimination between particles is done. Based on the fitness standards, social leaders are selected, and external archive pruning is performed as to how to select the best non-dominated particles in the archive.

## Experimental results and discussion

In the following section, the algorithms performance is discussed. The algorithms used for comparison are MaOPSO, NSGA III, MaOBAT and SMPSO [8]. These comparisons give three different aspect of how the convergence and diversity values are affected by using three different algorithms.

In the first aspect, the convergence of many objective algorithms is better than the multi-objective algorithms. In the second aspect, the analysis of the pareto dominance-based algorithms are not well converged towards PF in MaOPs. Well-known quality indicator such as inverted generational distance (IGD), generational distance (GD) and hypervolume (HV) are used to perform the analysis based on the diversity and convergence of the algorithms.

Experiments are performed with varying number of objectives (m) such that m = [2, 3, 5, 7, 10], on different algorithms such as MaOPSO, NSGA-III, MaOBAT, SMPSO and then each algorithm is evaluated with quality measure algorithm to get the qualitative analysis. Significance of the obtained results is also demonstrated using T-Test.

### Benchmark test problems

In this paper, DTLZ [2, 4, 6] problems are used as the benchmark problem [36]. The number of decision variables (n) and number of objectives (m) are altered during the experimentations. The formula is as follows: n = m+k+1. DTLZ4 is defined as a difficult problem regarding diversity. DTLZ6 is used to see the how well an algorithm can converge to a curve. We have used the value of k as 10 for all problems.

### Parameters Settings

#### MaOBAT

**Fig 1:**
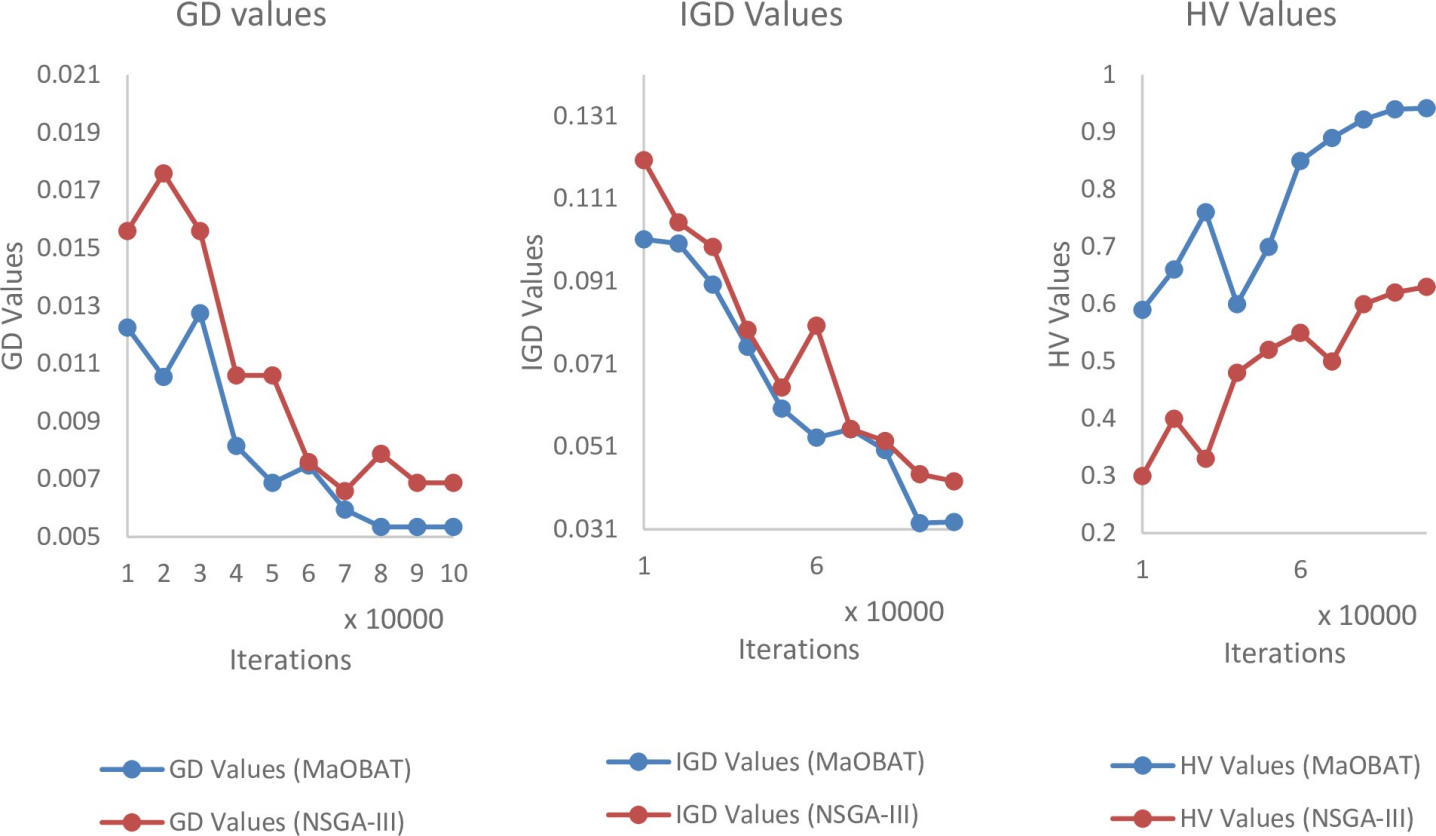
Comparison of GD (10 objectives), IGD (7 objectives) and HV values (7 objectives) of MaOBAT and NSGA-III on different number of iterations on DTLZ2.

**Fig 2:**
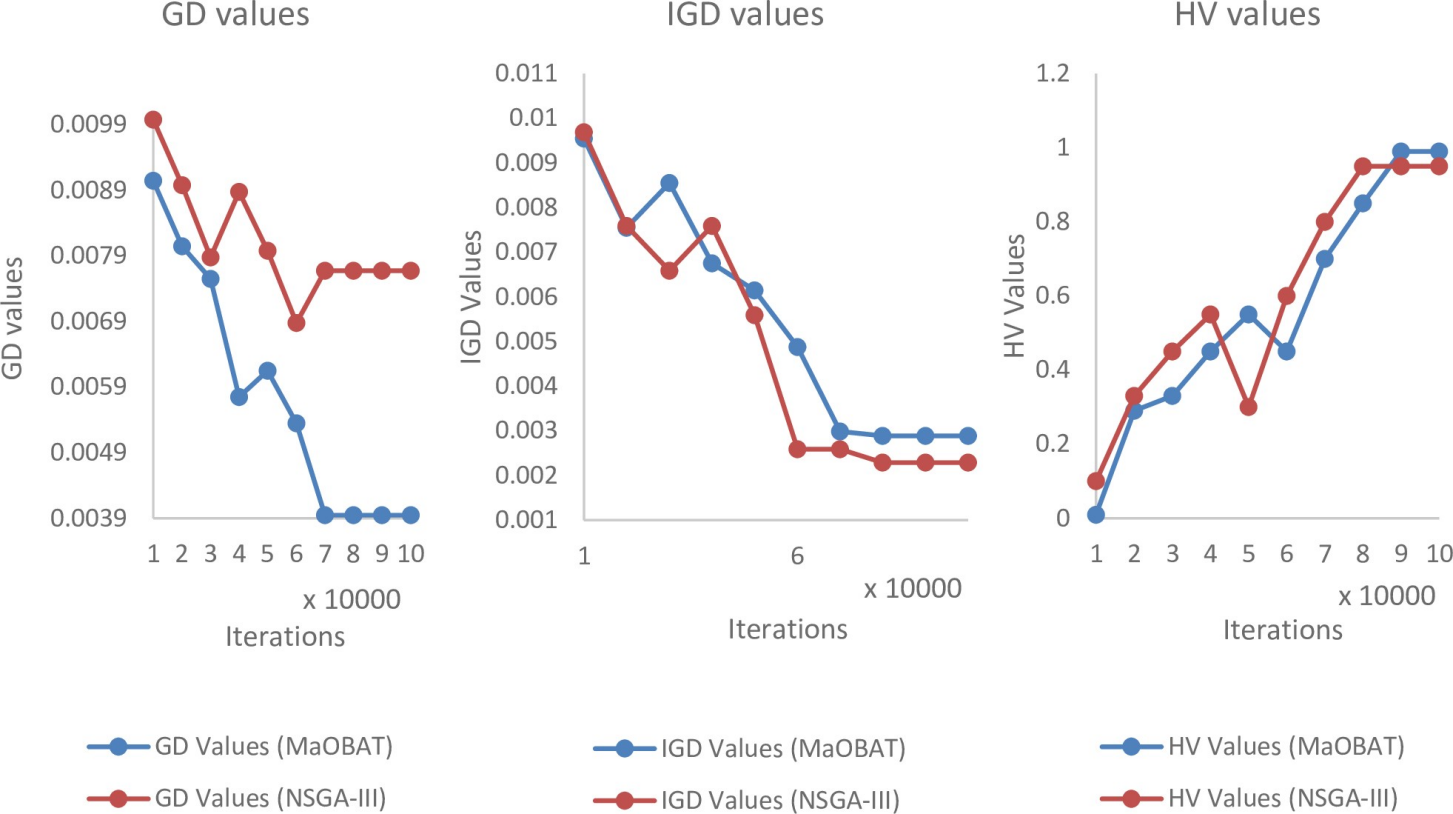
Comparison of GD, IGD and HV values of MaOBAT and NSGA-III on different number of iterations (10 objectives) on DTLZ4.

**Fig 3:**
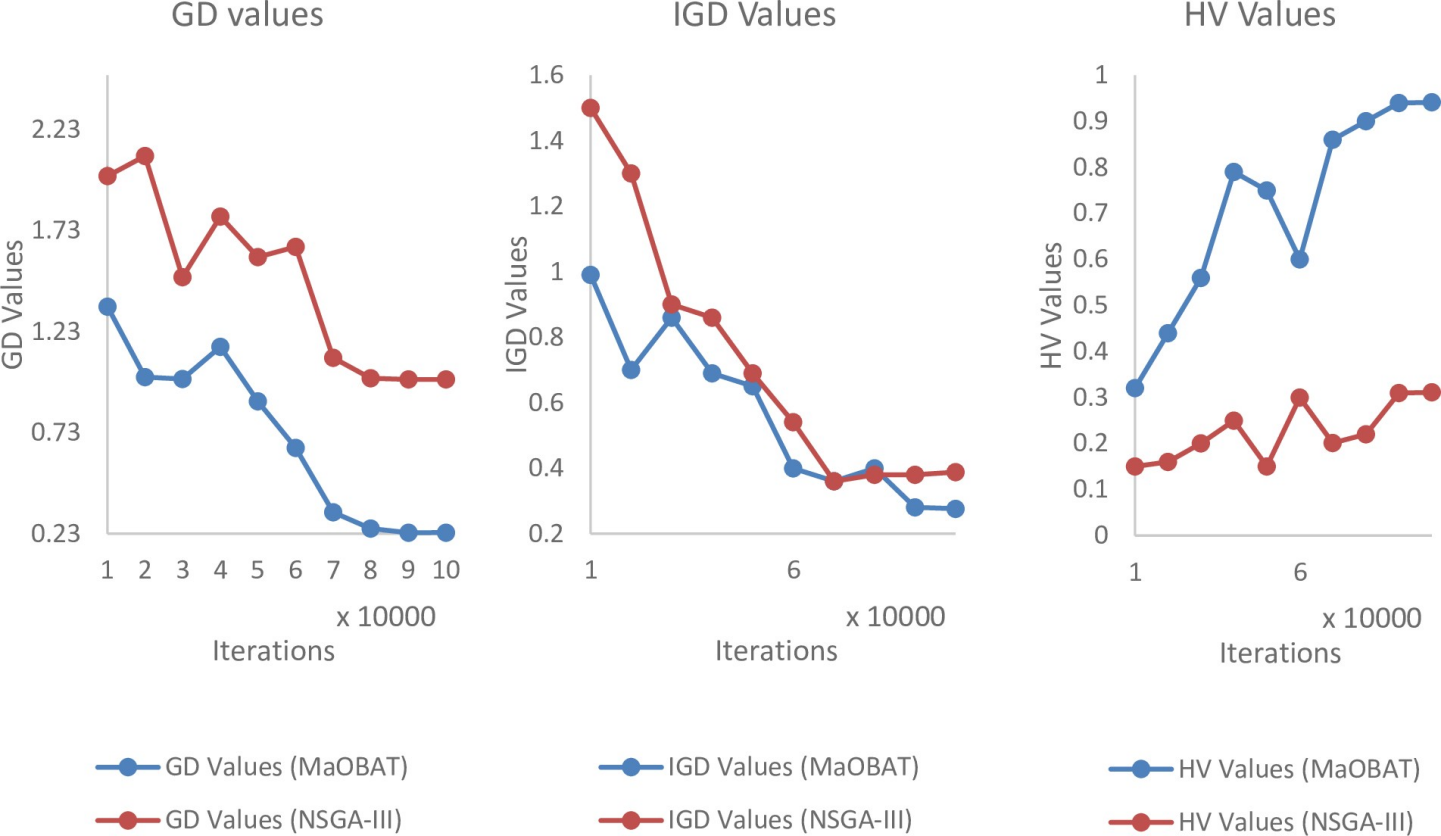
Comparison of GD, IGD and HV values of MaOBAT and NSGA-III on different number of iterations (10 objectives) on DTLZ6.

In MaOBAT algorithm, f_min_ and f_max_ are set to 0 and 1 respectively. Loudness is assigned the random value from [1,2] for each bat. Pulse emission rate (*α*) and loudness reduction (*γ*) are assigned values in between 0.7 to 0.9 and are used same as these values give optimal results [4]. The number of divisions in each objective is represented as p and m is the number of objectives and their values is used in the reference point calculation by this formula $H=c_{p}^{m+p-1}$. Generally, the greater the number of reference point means better results. The size of archive is dependent on the number of objectives as shown in Table 1. The archive size is based on the number of reference points but in case of 10 objectives we assign archive size equal to 500 as of computational limitations. For the experiments, 100,000 iterations are used, as the many objective algorithms takes some time to show its convergent behavior for each algorithm as shown in Figs 1, 2 and 3. MaOPSO [28] also executed 100,000 iterations to calculate the final Pareto Front because it demonstrated the convergent behavior until this number of iterations. The number of iterations is kept same for all the compared algorithms to have a fair comparison of the algorithms with MaOBAT. Moreover, the parameters’ settings in the selected algorithms are inspired from the MaOPSO and the values of the parameters were selected such that the aim was to achieve the convergence of the algorithms [28]. Mutation probability of (1/number of decision variable) is used, with mutation distribution of 20 while using polynomial mutation, according to Nebro et al [8].

**Table 1:** Parameters settings in the selected algorithms.

Objectives	p	Number of points	Archive size	Population size
2	200	200	200	100
3	20	231	231	100
5	8	495	495	100
7	6	924	924	200
10	5	2002	500	300

#### MaOPSO

In MaOPSO, the parameter values used are followed from the previous paper [28]. We run the algorithm for 100,000 iterations with different population size and reference point as shown in the Table 1.

#### NSGA-III

In NSGA III, the parameter values used are followed from MaOPSO [28]. We run the algorithm for 100,000 iterations with different population size and reference point as shown in the Table 1.

#### SMPSO

For SMPSO, the parameter values which are used here are according to the ones mentioned in MaOPSO [28]. The algorithm is run for 100,000 iterations to have the fair comparison with other algorithms. Mutation probability of (1/number of decision variable) is used, with mutation distribution of 20 while using polynomial mutation, according to Nebro et al [8].

#### Parametrical analysis

**Fig 4:**
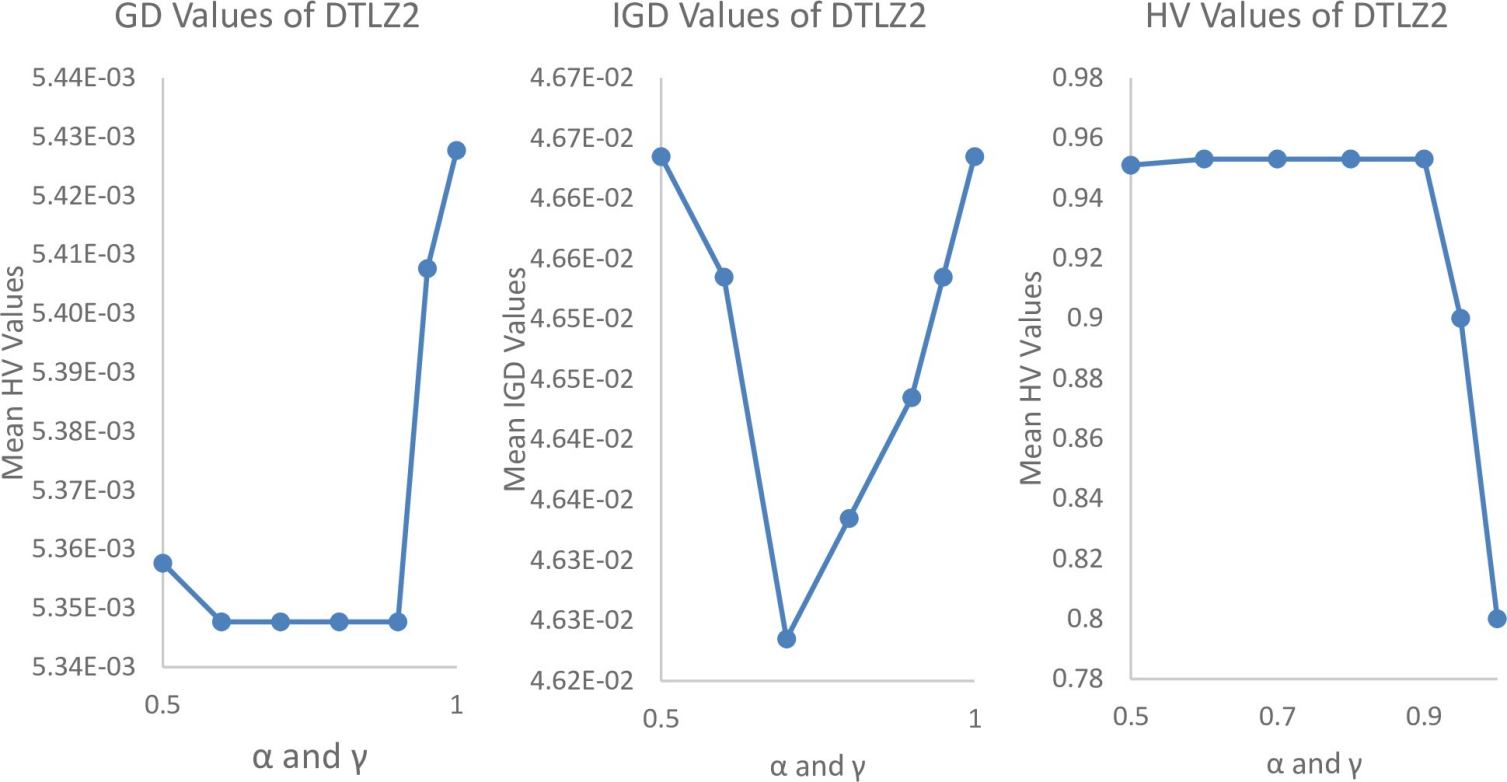
Parametrical analysis of α and γ with Iterations = 100000, m = 10, p = 5, N = 300, and archive size = 500, while considering GD values of MaOBAT of DTLZ2.

**Fig 5:**
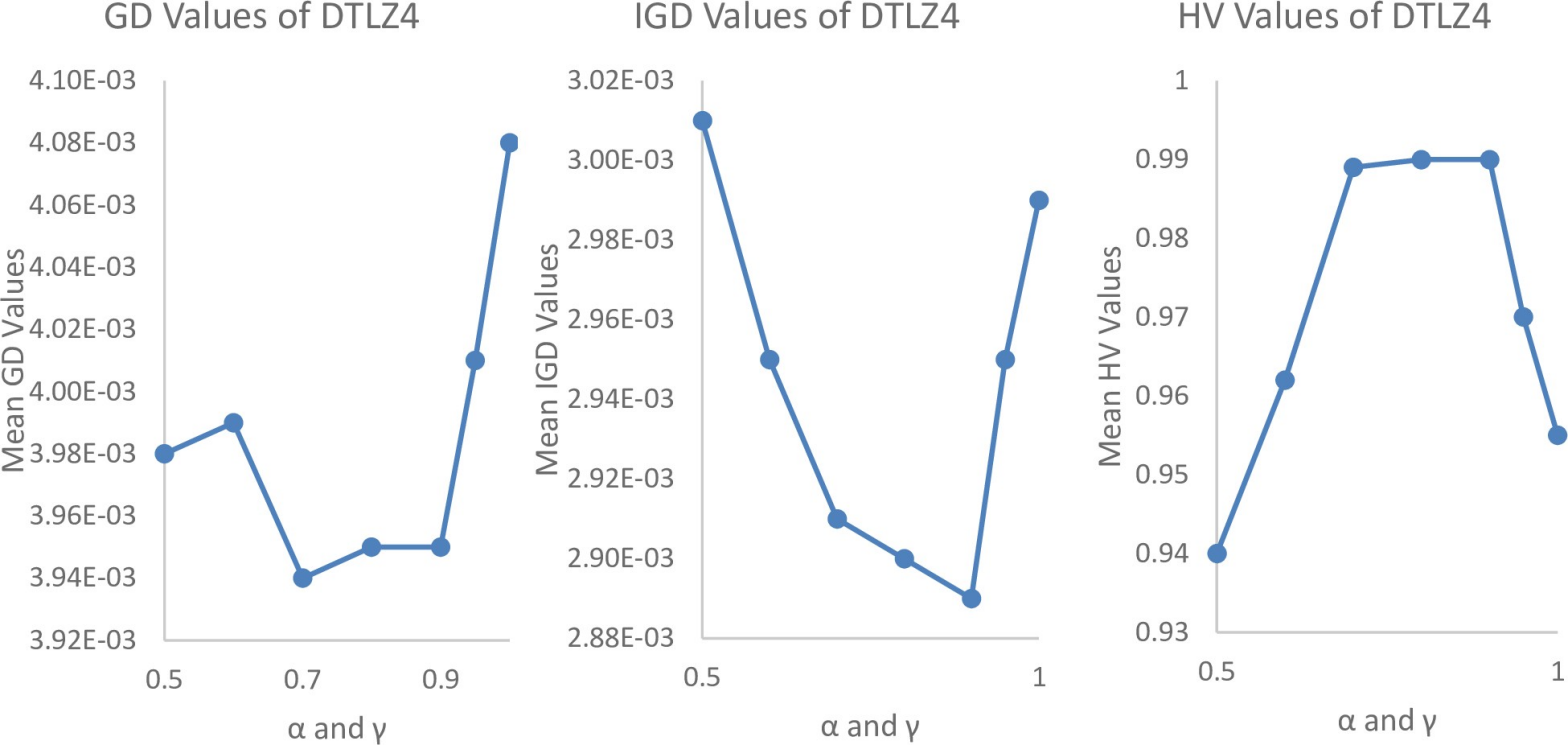
Parametrical analysis of α and γ with Iterations = 100000, m = 10, p = 5, N = 300, and archive size = 500, while considering GD values of MaOBAT of DTLZ4.

**Fig 6:**
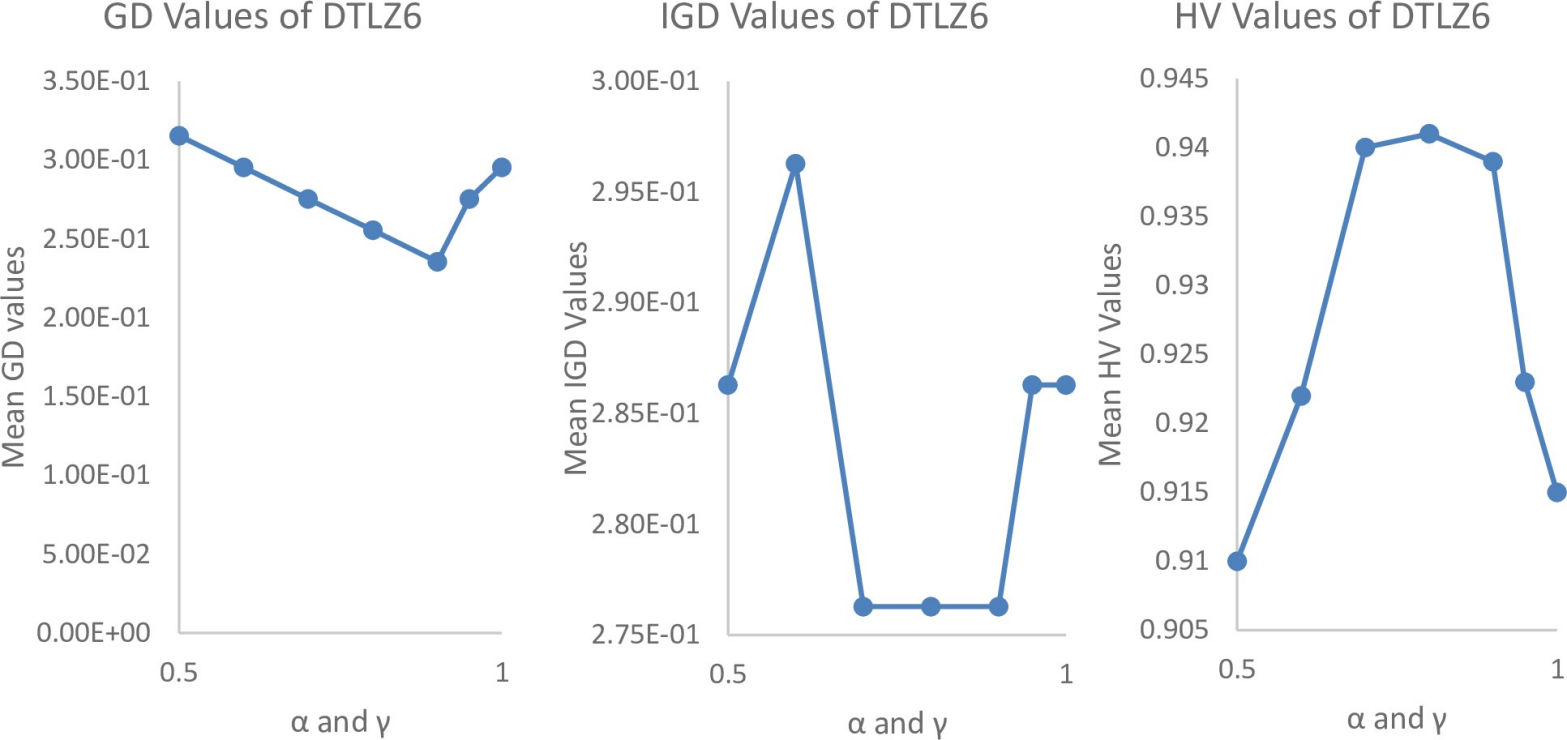
Parametrical analysis of α and γ with Iterations = 100000, m = 10, p = 5, N = 300, and archive size = 500, while considering GD values of MaOBAT of DTLZ6.

We have tested it using a different range of parameters such as Pulse emission rate (*α*) and loudness reduction (*γ*) and population size (n) as shown in Figs 4, 5 and 6. By varying *α* = 0.5, 0.6, 0.7, 0.8, 0.9, 0.95, 1 and *γ* = 0.5, 0.6, 0.7, 0.8, 0.9, 0.95, 1, 0, it is found that the best parameters for the most experiments are *α* = 0.7 to 0.9 and *γ* = 0.7 to 0.9.

### Computational results and discussion

In the following section, we analyze the value of the GD, IGD and HV for MaOBAT, NSGA-III, MaOPSO and SMPSO. The analysis shows that how these algorithms perform in accordance to convergence and diversity values. Tables 2–4 shows the values of the GD metrics; Tables 5–7 show the values of IGD metrics and Tables 8–10 shows the values of the HV metrics.

**Table 2:** GD metric of problem DTLZ2 (100,000 iterations). Values in bold are best values. ‘≈’, ‘−’ and ‘+’ show that the result is statistically similar, significantly worse and significantly better to that of MaOBAT, respectively (with alpha = 0.05).

Objectives	MaOPSO	NSGA III	MaOBAT	SMPSO
2	2.058336E-4 (3.25e-5) ≈	1.974504E-4 (5.06e-5) ≈	1.721903E-4 (6.00e-5)	**1.662371E-4 (2.99e-5)** ≈
3	3.843454E-4 (4.20e-5) ≈	**3.324329E-4 (3.95e-5)** ≈	3.514326E-4 (5.23e-5)	3.487237E-4 (4.22e-5) ≈
5	2.645245E-3 (2.82e-4) ≈	3.234534E-2 (3.02e-3) −	**2.562546E-3 (3.50e-4)**	3.476738E-2 (3.66e-3) −
7	4.876875E-3 (3.01e-4) −	**3.254657E-3 (3.65e-4) +**	3.765876E-3 (4.20e-4)	3.639874E-3 (2.98e-4) ≈
10	5.546757E-3 (3.99e-4) ≈	6.876875E-3 (2.99e-4) −	**5.347683E-3 (2.98e-4)**	7.874293E-3 (2.66e-4) −

**Table 3:** GD metric of problem DTLZ4 (100,000 iterations). Values in bold are best values. ‘≈’, ‘−’ and ‘+’ show that the result is statistically similar, significantly worse and significantly better to that of MaOBAT, respectively (with alpha = 0.05).

Objectives	MaOPSO	NSGA III	MaOBAT	SMPSO
2	7.653915E-5 (1.29e-6) −	8.258954E-5 (1.02e-6) −	**7.241409E-5 (1.33e-6)**	8.354345E-5 (1.69e-6) −
3	4.634534E-4 (3.35e-5) −	4.644548E-4 (1.98e-5) −	**4.352342E-4 (2.36e-6)**	4.735345E-4 (1.95e-5) −
5	4.284792E-3 (7.66e-4) ≈	7.456634E-3 (6.99e-4) −	**3.826273E-3 (3.11e-4)**	7.887236E-3 (5.57e-4) −
7	3.836576E-3 (8.36e-4) ≈	8.546557E-3 (5.96e-4) −	**3.826273E-3 (4.52e-4)**	9.123127E-3 (5.65e-4) −
10	3.987687E-3 (6.35e-4) ≈	7.674298E-3 (4.66e-4) −	**3.946776E-3 (4.45e-4)**	8.642342E-3 (4.20e-4) −

**Table 4:** GD metric of Problem DTLZ6 (100,000 iterations). Values in bold are best values. ‘≈’, ‘−’ and ‘+’ show that the result is statistically similar, significantly worse and significantly better to that of MaOBAT, respectively (with alpha = 0.05).

Objectives	MaOPSO	NSGA III	MaOBAT	SMPSO
2	2.523425E-3 (9.74e-4) ≈	2.624323E-3 (6.41e-4) ≈	**2.501345E-3 (6.17e-4)**	2.523498E-3 (1.18e-5) ≈
3	1.274898E-2 (5.27e-3) ≈	1.275687E-2 (9.39e-3) ≈	**1.178726E-2 (6.20e-3)**	1.234532E-2 (1.18e-4) ≈
5	7.242343E-2 (1.38e-3) −	8.352435E-2 (1.66e-3) −	**7.123423E-2 (1.05e-4)**	8.653453E-2 (2.51e-3) −
7	1.724637E-1 (1.87e-2) −	0.84872638 (2.20e-2) −	**1.464564E-1 (1.93e-2)**	0.99873468 (2.10e-2) −
10	2.345345E-1 (1.78e-2) ≈	0.9934984 (4.01e-2) −	**2.354565E-1 (2.65e-2)**	1.0473486 (3.12e-2) −

**Table 5:** IGD metric of Problem DTLZ2 (100,000 iterations). Values in bold are best values. ‘≈’, ‘−’ and ‘+’ show that the result is statistically similar, significantly worse and significantly better to that of MaOBAT, respectively (with alpha = 0.05).

Objectives	MaOPSO	NSGA III	MaOBAT	SMPSO
2	2.070280E-4 (4.29e-5) ≈	**1.125479E-4 (4.04e-5) +**	2.356600E-4 (5.73e-5)	1.629348E-4 (3.96e-5) +
3	**1.345434E-3 (4.39e-4)** ≈	1.434534E-3 (4.06e-4) ≈	1.545342E-3 (4.56e-4)	1.647298E-3 (3.90e-4) ≈
5	2.634534E-2 (3.18e-3) +	**2.145345E-2 (3.09e-3) +**	3.245235E-2 (3.41e-3)	2.423423E-2 (3.12e-3) +
7	3.376576E-2 (2.72e-3) ≈	4.265467E-2 (3.93e-3) −	**3.254655E-2 (3.98e-3)**	3.534532E-2 (3.17e-3) ≈
10	**4.353847E-2 (3.08e-3)** ≈	4.734879E-2 (3.48e-3) ≈	4.623487E-2 (3.13e-3)	4.823488E-2 (2.33e-3) ≈

**Table 6:** IGD metric of Problem DTLZ4 (100,000 iterations). Values in bold are best values. ‘≈’, ‘−’ and ‘+’ show that the result is statistically similar, significantly worse and significantly better to that of MaOBAT, respectively (with alpha = 0.05).

Objectives	MaOPSO	NSGA III	MaOBAT	SMPSO
2	**7.465870E-5 (1.19e-6) +**	8.543565E-5 (1.09e-6) +	8.965765E-5 (6.66e-7)	8.629348E-5 (1.27e-6) +
3	7.465870E-5 (2.95e-6) −	7.465860E-5 (1.65e-6) −	**7.273498E-4 (9.25e-7)**	9.749284E-5 (1.59e-6) −
5	9.487234E-3 (6.94e-4) −	7.423453E-3 (6.53e-4) −	**6.387462E-3 (3.39e-4)**	7.624232E-3 (6.61e-4) −
7	1.468879E-3 (7.84e-4) ≈	1.687987E-3 (5.87e-4) ≈	**1.265476E-3 (5.34e-4)**	0.5487328 (6.61e-4) −
10	2.539875E-3 (5.32e-4) ≈	**2.287428E-3 (4.74e-4) +**	2.887493E-3 (4.90e-4)	0.7648237 (3.56e-4) −

**Table 7:** IGD metric of Problem DTLZ6 (100,000 iterations). Values in bold are best values. ‘≈’, ‘−’ and ‘+’ show that the result is statistically similar, significantly worse and significantly better to that of MaOBAT, respectively (with alpha = 0.05).

Objectives	MaOPSO	NSGA III	MaOBAT	SMPSO
2	4.312121E-2 (5.63e-4) −	3.234234E-2 (2.07e-3) −	**2.123423E-2 (3.20e-3)**	3.423422E-2 (4.54e-4) −
3	4.876768E-2 (5.89e-3) −	5.328798E-2 (2.05e-3) −	4.283915E-2 (3.80e-3)	**1.345332E-2 (3.11e-3) +**
5	**5.384500E-2 (1.08e-3)** ≈	6.123093E-2 (2.48e-4) −	5.873298E-2 (2.05e-4)	5.934223E-2 (3.36e-4) −
7	1.388475E-1 (7.19e-2) ≈	1.398768E-1 (2.50e-2) ≈	**1.378698E-1 (3.50e-2)**	1.414823E-1 (1.58e-2) ≈
10	3.287498E-1 (1.27e-2) −	3.876987E-1 (1.38e-2) −	**2.762847E-1 (1.33e-2)**	3.288768E-1 (1.34e-2) −

**Table 8:** HV metric of Problem DTLZ2 (100,000 iterations). Values in bold are best values. ‘≈’, ‘−’ and ‘+’ show that the result is statistically similar, significantly worse and significantly better to that of MaOBAT, respectively (with alpha = 0.05).

Objectives	MaOPSO	NSGA III	MaOBAT	SMPSO
2	0.990 (0.014060) **≈**	**0.992 (0.026770) ≈**	0.991 (0.010238)	0.962 (0.011431) −
3	**0.989 (0.021569) +**	0.837 (0.007659) −	0.893 (0.011332)	0.862 (0.010369) −
5	**0.990 (0.012970) +**	0.600 (0.006409) −	0.871 (0.012202)	0.743 (0.011174) −
7	0.931 (0.011988) **≈**	0.630 (0.015702) −	**0.942 (0.017528)**	0.771 (0.019081) −
10	0.951 (0.017303) **≈**	0.480 (0.010778) −	**0.953 (0.008167)**	0.420 (0.008177) −

**Table 9:** HV metric of Problem DTLZ4 (100,000 iterations). Values in bold are best values. ‘≈’, ‘−’ and ‘+’ show that the result is statistically similar, significantly worse and significantly better to that of MaOBAT, respectively (with alpha = 0.05).

Objectives	MaOPSO	NSGA III	MaOBAT	SMPSO
2	0.901 (0.007350) −	**0.983 (0.013268) +**	0.952 (0.003427)	0.988 (0.010474) +
3	0.921 (0.010605) ≈	0.854 (0.010401) −	**0.933 (0.013729)**	0.849 (0.011190) −
5	0.983 (0.014543) ≈	0.780 (0.013750) −	**0.992 (0.008497)**	0.701 (0.007513) −
7	0.992 (0.007072) ≈	0.840 (0.017627) −	**0.993 (0.014843)**	0.700 (0.015733) −
10	0.989 (0.019326) ≈	0.950 (0.008873) −	**0.990 (0.009701)**	0.820 (0.014723) −

**Table 10:** HV metric of Problem DTLZ6 (100,000 iterations). Values in bold are best values. ‘≈’, ‘−’ and ‘+’ show that the result is statistically similar, significantly worse and significantly better to that of MaOBAT, respectively (with alpha = 0.05).

Objectives	MaOPSO	NSGA III	MaOBAT	SMPSO
2	0.990 (0.014324) ≈	0.801 (0.024760) −	**0.992 (0.004303)**	0.911 (0.013035) −
3	0.557 (0.014017) −	0.931 (0.022242) −	**0.993 (0.007557)**	0.972 (0.009004) −
5	0.621 (0.011132) −	0.382 (0.018306) −	**0.992 (0.007555)**	0.971 (0.016300) −
7	0.940 (0.009473) ≈	0.401 (0.008810) −	0.945 (0.004768)	**0.964 (0.007558) +**
10	0.930 (0.014989) ≈	0.311 (0.033148) −	**0.941 (0.007621)**	0.931 (0.011066) ≈

For **DTLZ 2** (Tables 2, 5 and 8). In case of convergence, MaOBAT performs better when objectives are 5 and 10 as shown in Table 2 (For GD values). In regard to IGD, the MaOBAT performs better and better as the number of objectives increased as shown in Table 5. With HV consideration, MaOBAT shows the same behavior as shown in the IGD as it performs better when objectives are increased as shown in Table 8.

For **DTLZ 4** (Tables 3, 6 and 9), using all the quality indicators (GD, IGD, HV). MaOBAT performs better or significantly better (at 95% confidence interval) for the most number of experiments for all instances of this problem. So, MaOBAT give the best compromise between diversity and convergence of this problem.

For **DTLZ 6** (Tables 4, 7 and 10), MaOBAT performs better or significantly better (at 95% confidence interval) for the most number of experiments showing the good convergence. When IGD values are considered, the MaOBAT again performed significantly better (at 95% confidence interval) for different number of objectives. On the other hand, when considering the HV, the MaOBAT performs significantly better (at 95% confidence interval) than the other many objective algorithms at most times.

The combination of echolocation with swarm intelligence and search process enhances the properties of swarm-based algorithms so this thing makes BAT algorithm little more effective than the swarm-based algorithms in some of the scenarios.

## Conclusion and future work

This paper proposes a many objective bat algorithm (MaOBAT) to solve many-objective optimization problems by effectively approximating the Pareto approximation (PA) with high diversity and good convergence. In the proposed method, the reference-based approach in MOBAT is used and MaOBAT is proposed, as background study shows that this approach is being used effectively for MaOPs. Moreover, in the fitness assignment method, the reference points are evenly distributed for imposing the selection pressure for getting the good convergence against the true PF. The experimental results demonstrate that the MaOBAT have good capability in generating the PF with good convergence and high diversity. Significance of the obtained results is also demonstrated using T-Test. Empirical study shows that MaOBAT works efficiently in comparison to MaOPSO, SMPSO and NSGA III. Moreover, it computes high diversity and good convergence in a reasonable time. To maintain pressure selection towards PF and to get the high diversity, extreme solutions and uniformly distributed reference set are used. Furthermore, the experimental results demonstrate that the MaOBAT algorithm has significant advantages over several state-of-the-art many objective algorithms in terms of the excellence of the solution.

In future, more experiments can be done for different benchmark problems, specifically for practical problems having high dimensionality and complex PF to get more meticulous performance analysis of the MaOBAT.

## References

[1] Coello CA, Lamont GB, Van Veldhuizen DA. Evolutionary algorithms for solving multi-objective problems New York: Springer; 2007 9 18.

[2] Zhou A, Qu BY, Li H, Zhao SZ, Suganthan PN, Zhang Q. Multiobjective evolutionary algorithms: A survey of the state of the art. Swarm and Evolutionary Computation. 2011 3 1;1(1):32–49.

[3] Reyes-Sierra M, Coello CC. Multi-objective particle swarm optimizers: A survey of the state-of-the-art. International journal of computational intelligence research. 2006 3 22;2(3):287–308.

[4] Yang XS. Bat algorithm for multi-objective optimisation. arXiv preprint arXiv:1203.6571. 2012 3 29.

[5] Ishibuchi H, Tsukamoto N, Nojima Y. Evolutionary many-objective optimization: A short review. In2008 IEEE Congress on Evolutionary Computation (IEEE World Congress on Computational Intelligence) 2008 6 1 (pp. 2419–2426). IEEE.

[6] Deb K, Pratap A, Agarwal S, Meyarivan TA. A fast and elitist multiobjective genetic algorithm: NSGA-II. IEEE transactions on evolutionary computation. 2002 8 7;6(2):182–97.

[7] Zitzler E, Laumanns M, Thiele L. SPEA2: Improving the strength Pareto evolutionary algorithm. TIK-report. 2001;103.

[8] Nebro AJ, Durillo JJ, Garcia-Nieto J, Coello CC, Luna F, Alba E. SMPSO: A new PSO-based metaheuristic for multi-objective optimization. In2009 IEEE Symposium on Computational Intelligence in Multi-Criteria Decision-Making (MCDM) 2009 3 30 (pp. 66–73). IEEE.

[9] Cai L, Qu S, Yuan Y, Yao X. A clustering-ranking method for many-objective optimization. Applied Soft Computing. 2015 10 1;35:681–94.

[10] Wagner T, Beume N, Naujoks B. Pareto-, aggregation-, and indicator-based methods in many-objective optimization InInternational conference on evolutionary multi-criterion optimization 2007 3 5 (pp. 742–756). Springer, Berlin, Heidelberg.

[11] Wang R, Purshouse RC, Fleming PJ. Preference-inspired coevolutionary algorithms for many-objective optimization. IEEE Transactions on Evolutionary Computation. 2012 6 11;17(4):474–94.

[12] Farina M, Amato P. A fuzzy definition of" optimality" for many-criteria optimization problems. IEEE Transactions on Systems, Man, and Cybernetics-Part A: Systems and Humans. 2004 4 19;34(3):315–26.

[13] Yang S, Li M, Liu X, Zheng J. A grid-based evolutionary algorithm for many-objective optimization. IEEE Transactions on Evolutionary Computation. 2013 1 1;17(5):721–36.

[14] Li B, Li J, Tang K, Yao X. Many-objective evolutionary algorithms: A survey. ACM Computing Surveys (CSUR). 2015 9 29;48(1):1–35.

[15] Garza-Fabre M, Toscano-Pulido G, Coello CA. Two novel approaches for many-objective optimization. InIEEE Congress on Evolutionary Computation 2010 7 18 (pp. 1–8). IEEE.

[16] Mostaghim S, Schmeck H. Distance based ranking in many-objective particle swarm optimization InInternational Conference on Parallel Problem Solving from Nature 2008 9 13 (pp. 753–762). Springer, Berlin, Heidelberg.

[17] Purshouse RC, Fleming PJ. On the evolutionary optimization of many conflicting objectives. IEEE transactions on evolutionary computation. 2007 11 27;11(6):770–84.

[18] Ishibuchi H, Sakane Y, Tsukamoto N, Nojima Y. Evolutionary many-objective optimization by NSGA-II and MOEA/D with large populations. In2009 IEEE International Conference on Systems, Man and Cybernetics 2009 10 11 (pp. 1758–1763). IEEE.

[19] Cheng J, Yen GG, Zhang G. A many-objective evolutionary algorithm with enhanced mating and environmental selections. IEEE Transactions on Evolutionary Computation. 2015 4 21;19(4):592–605.

[20] Ishibuchi H, Tsukamoto N, Hitotsuyanagi Y, Nojima Y. Effectiveness of scalability improvement attempts on the performance of NSGA-II for many-objective problems. InProceedings of the 10th annual conference on Genetic and evolutionary computation 2008 7 12 (pp. 649–656).

[21] Asafuddoula M, Ray T, Sarker R. A decomposition-based evolutionary algorithm for many objective optimization. IEEE Transactions on Evolutionary Computation. 2014 7 16;19(3):445–60.

[22] Ishibuchi H, Sakane Y, Tsukamoto N, Nojima Y. Simultaneous use of different scalarizing functions in MOEA/D. InProceedings of the 12th annual conference on Genetic and evolutionary computation 2010 7 7 (pp. 519–526).

[23] GriYn DR, Webster FA, Michael CR. The echolocation of Xying insects by bats. Anim Behav. 1960;8:3–4.

[24] Talal R. Comparative study between the (ba) algorithm and (pso) algorithm to train (rbf) network at data classification. International Journal of Computer Applications. 2014 1 1;92(5):16–22.

[25] Khan K, Sahai A. A comparison of BA, GA, PSO, BP and LM for training feed forward neural networks in e-learning context. International Journal of Intelligent Systems and Applications. 2012 6 1;4(7):23.

[26] Jaddi NS, Abdullah S, Hamdan AR. Optimization of neural network model using modified bat-inspired algorithm. Applied Soft Computing. 2015 12 1;37:71–86.

[27] Deb K, Jain H. An evolutionary many-objective optimization algorithm using reference-point-based nondominated sorting approach, part I: solving problems with box constraints. IEEE transactions on evolutionary computation. 2013 9 16;18(4):577–601.

[28] Figueiredo EM, Ludermir TB, Bastos-Filho CJ. Many objective particle swarm optimization. Information Sciences. 2016 12 20;374:115–34.

[29] Cui Z, Chang Y, Zhang J, Cai X, Zhang W. Improved NSGA-III with selection-and-elimination operator. Swarm and Evolutionary Computation. 2019 9 1; 49:23–33.

[30] Apornak A, Raissi S, Keramati A, Khalili-Damghani K. Optimizing human resource cost of an emergency hospital using multi-objective Bat algorithm. International Journal of Healthcare Management. 2020 1 4:1–7.

[31] Raychaudhuri A, De D. Bio-inspired Algorithm for Multi-objective Optimization in Wireless Sensor Network In Nature Inspired Computing for Wireless Sensor Networks 2020 (pp. 279–301). Springer, Singapore.

[32] Liu TY. Learning to rank for information retrieval. Foundations and Trends® in Information Retrieval. 2009 6 27;3(3):225–331.

[33] Li J, Liu G, Yan C, Jiang C. Robust learning to rank based on portfolio theory and AMOSA algorithm. IEEE Transactions on Systems, Man, and Cybernetics: Systems. 2016 7 22;47(6):1007–18.

[34] Li X, Han S, Zhao L, Gong C, Liu X. New dandelion algorithm optimizes extreme learning machine for biomedical classification problems. Computational intelligence and neuroscience. 2017;2017. 10.1155/2017/4523754 PMC5612329 29085425

[35] Zhu H, Liu G, Zhou M, Xie Y, Kang Q. Dandelion Algorithm With Probability-Based Mutation. IEEE Access. 2019 7 9;7:97974–85.

[36] Deb K, Thiele L, Laumanns M, Zitzler E. Scalable test problems for evolutionary multiobjective optimization InEvolutionary multiobjective optimization 2005 (pp. 105–145). Springer, London.

[37] Kirkpatrick S, Gelatt CD, Vecchi MP. Optimization by simulated annealing. science. 1983 5 13;220(4598):671–80. 10.1126/science.220.4598.671 17813860

[38] Kung HT, Luccio F, Preparata FP. On finding the maxima of a set of vectors. Journal of the ACM (JACM). 1975 10 1;22(4):469–76.

[39] Yang XS. Nature-inspired metaheuristic algorithms Luniver press; 2010.

[40] Younas I, Kamrani F, Bashir M, Schubert J. Efficient genetic algorithms for optimal assignment of tasks to teams of agents. Neurocomputing. 2018 11 7;314:409–28.

[41] Geem ZW, Kim JH, Loganathan GV. A new heuristic optimization algorithm: harmony search. simulation. 2001 2;76(2):60–8.

[42] Kaveh A, Talatahari S. A novel heuristic optimization method: charged system search. Acta Mechanica. 2010 9 1;213(3–4):267–89.

[43] Jaddi NS, Abdullah S, Hamdan AR. Multi-population cooperative bat algorithm-based optimization of artificial neural network model. Information Sciences. 2015 Feb 10;294:628–44.

[44] Kennedy J, Eberhart R. Particle swarm optimization. InProceedings of ICNN'95-International Conference on Neural Networks 1995 11 27 (Vol. 4, pp. 1942–1948). IEEE.

[45] Fister I, Fong S, Brest J. A novel hybrid self-adaptive bat algorithm. The Scientific World Journal. 2014;2014. 10.1155/2014/709738 PMC4000672 25187904

[46] Rao SS. Engineering optimization: theory and practice John Wiley & Sons; 2019 11 12.

